# Metagenomics of Coral Reefs Under Phase Shift and High Hydrodynamics

**DOI:** 10.3389/fmicb.2018.02203

**Published:** 2018-10-04

**Authors:** Pedro Milet Meirelles, Ana Carolina Soares, Louisi Oliveira, Luciana Leomil, Luciana Reis Appolinario, Ronaldo Bastos Francini-Filho, Rodrigo Leão de Moura, Renato Tenan de Barros Almeida, Paulo S. Salomon, Gilberto Menezes Amado-Filho, Ricardo Kruger, Eduardo Siegle, Diogo A. Tschoeke, Isao Kudo, Sayaka Mino, Tomoo Sawabe, Cristiane C. Thompson, Fabiano L. Thompson

**Affiliations:** ^1^Institute of Biology and SAGE-COPPE, Federal University of Rio de Janeiro, Rio de Janeiro, Brazil; ^2^Department of Environment and Engineering, Federal University of Paraíba, Rio Tinto, Brazil; ^3^Rio de Janeiro Botanical Garden Research Institute (IP-JBRJ), Rio de Janeiro, Brazil; ^4^Department of Cellular Biology, University of Brasília, Brasília, Brazil; ^5^Oceanographic Institute, University of São Paulo, São Paulo, Brazil; ^6^Graduate School of Environmental Science, Hokkaido University, Sapporo, Japan; ^7^Laboratory of Microbiology, Faculty of Fisheries Sciences, Hokkaido University, Hakodate, Japan

**Keywords:** coral reefs, microbialization, local stressors, early warning signals, metagenomics, resistence, hydrodynamics

## Abstract

Local and global stressors have affected coral reef ecosystems worldwide. Switches from coral to algal dominance states and microbialization are the major processes underlying the global decline of coral reefs. However, most of the knowledge concerning microbialization has not considered physical disturbances (e.g., typhoons, waves, and currents). Southern Japan reef systems have developed under extreme physical disturbances. Here, we present analyses of a three-year investigation on the coral reefs of Ishigaki Island that comprised benthic and fish surveys, water quality analyses, metagenomics and microbial abundance data. At the four studied sites, inorganic nutrient concentrations were high and exceeded eutrophication thresholds. The dissolved organic carbon (DOC) concentration (up to 233.3 μM) and microbial abundance (up to 2.5 × 10^5^ cell/mL) values were relatively high. The highest vibrio counts coincided with the highest turf cover (∼55–85%) and the lowest coral cover (∼4.4–10.2%) and fish biomass (0.06 individuals/m^2^). Microbiome compositions were similar among all sites and were dominated by heterotrophs. Our data suggest that a synergic effect among several regional stressors are driving coral decline. In a high hydrodynamics reef environment, high algal/turf cover, stimulated by eutrophication and low fish abundance due to overfishing, promote microbialization. Together with crown-of-thorns starfish (COTS) outbreaks and possible of climate changes impacts, theses coral reefs are likely to collapse.

## Introduction

Coral reefs are subjected to cyclic and episodic natural disturbances (e.g., storms), but their recovery potential is affected by interacting factors that vary regionally and at ocean basin scales (Roff and Mumby, 2012). For instance, in contrast with Pacific Ocean reefs, the lower resilience of Caribbean reefs is attributed to: (i) higher macroalgae recruitment and growth rates; (ii) basin-wide iron-enrichment from aeolian dust; (iii) the low diversity and abundance of fast-growing branching corals (e.g., Acroporidae) and herbivores; (iv) missing groups of herbivores (Roff and Mumby, 2012). The role of microbes in reef health is still under-acknowledged and represents an emerging frontier to a full understanding of coral reef resilience (Knowlton and Jackson, 2008; Garren and Azam, 2012). It is also not well understood how high hydrodynamics may affect reef microbial diversity.

The process by which a given marine ecosystem trophic structure shifts toward a higher microbial biomass and energy use is called “Microbialization” (Jackson et al., 2001). In coral reefs, the main possible causes of this process are overfishing and eutrophication, which act together in a positive feedback loop, increasing DOC concentrations, coral disease incidence, algal cover, and microbial abundance [DDAM model, see (Haas et al., 2016)]. Microbialization is positively correlated with human impacts across subregional (Bruce et al., 2012), ocean basin (McDole et al., 2012) and global spatial scales (Haas et al., 2016). Human influences also drive microbial energy use in the water column at Pacific coral reefs. The autotrophic activity alters energy fluxes at less impacted sites, increasing resistance mechanisms (McDoleSomera et al., 2016). However, the microbial abundance and metagenomic diversity are not well known in high hydrodynamic reefs, such as those in Ishigaki (Okinawa). These reefs experienced intense and chronic coral stress due to frequent typhoons which can became catastrophic with global warming (Hongo et al., 2012; Harii et al., 2014).

Japanese coral reefs are marine biodiversity hotspots with elevated endemism levels conditioned by non-reversing currents flowing from tropical to temperate latitudes (Roberts et al., 2002). Strong storms and typhoons are the foremost natural disturbance agents in the Okinawa region, where Japanese reefs are concentrated (Japanese Coral Reef Society, 2004). However, mass coral mortalities following thermal anomalies (Nishihira and Yamazato, 1974; Stone et al., 1999; Loya et al., 2001; Kayanne et al., 2002; Goto et al., 2010; Dadhich et al., 2012), crown-of-thorns starfish (COTS) outbreaks (Nishihira and Yamazato, 1974), and pollutant and nutrient runoffs (West and van Woesik, 2001; Kitada et al., 2008) are also escalating. Overfishing is a major feature of Southern Japan reefs (Liu et al., 2009). However, the microbial diversity in these reefs is not well documented.

Here, we investigated the metagenomic microbial diversity of the Ishigaki reefs, Okinawa, through a holistic approach. We incorporated data from benthic and fish assemblage assessments, water quality measurements (physical, chemical, biological, and microbiological parameters) and seawater metagenomics over three consecutive years (2012–2014) to investigate the following hypotheses: (I) eutrophication is leading coral to algal dominance states and (II) microbialization is an ongoing process and is more prevalent at high hydrodynamic reefs. Increased turf-algae dominance, low fish abundance, high nutrients, and DOC concentration, as well as heterotrophic microbial abundance, suggests a possible microbialization of Ishigaki reefs.

## Results

### Wave Modeling

**FIGURE 1:**
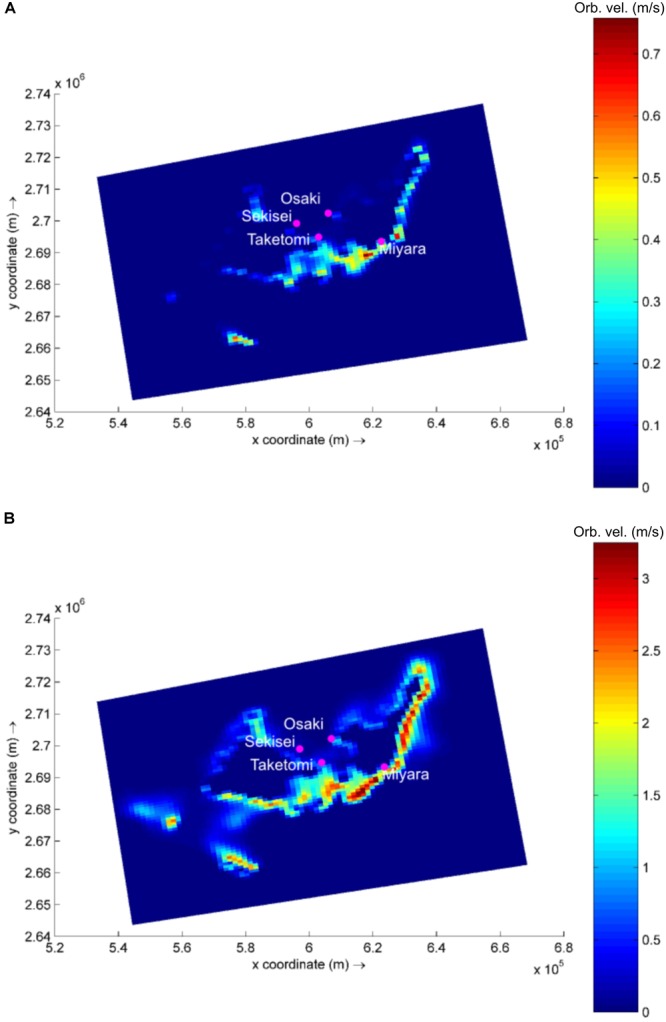
Modeled wave generated near bottom orbital velocity (m/s) in the area of interest. Example for calm **(A)** and typhoon **(B)** wave conditions. Note difference in color bar scales.

Based on the global wave generation model WaveWatch III (NOAA/NCEP), easterly waves dominate the study region (53% of occurrence), with heights ranging from 0.5 to 2.0 m (45% of occurrence). Waves reach the studied sites with varying power, and the exposed site is Miyara, followed by Osaki, Sekisei, and Taketomi (**Supplementary Table S13** and **Figure 1**). In a simulation of typhoon conditions, with an offshore wave height of 12 m combined with winds of up to 200 km/h (similar to the conditions observed during Super Typhoon Soulik, 2013) wave power increases by approx. 11,000 to 13,000% at Sekisei, Osaki, and Miyara. Typhoon conditions at Taketomi result in a lower increase in wave power, with an increase of approx. 4,000% (**Figure 1** and **Supplementary Table S13**).

Due to their positions, which protect them from incoming easterly waves, Taketomi and Sekisei present the lowest wave power levels for the tested conditions. Although protected from direct wave incidences, Sekisei still presents a high increase in wave power during typhoons, since the incoming swell and local wind action result in higher waves in Sekisei than in Taketomi. The high hydrodynamics also appear to contribute to the dispersion of microbes and coral disease among reefs. The high hydrodynamics may be considered an extra level of complexity while addressing coral reef microbialization and coral disease, not accounted for in previous studies.

### Benthic Cover

**FIGURE 2:**
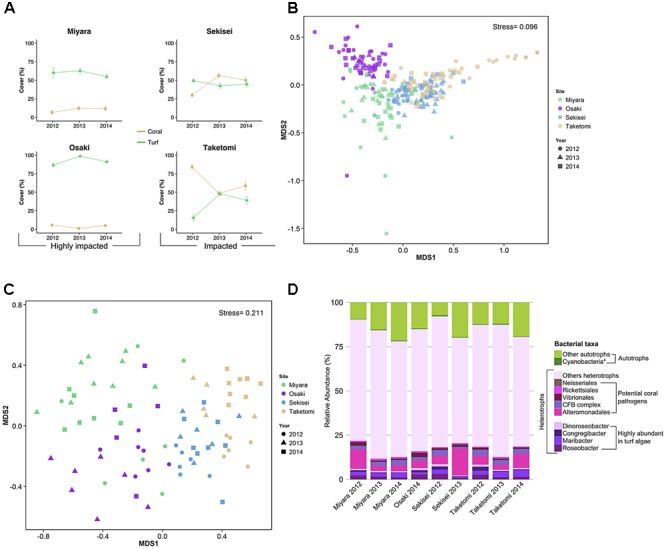
Coral reefs from Ishigaki Island are impacted at different levels. Yellow and green lines represent coral and algal cover in percentages for the reef sites and years, respectively **(A)**. The first two axes of non-metric multidimensional scaling (nMDS) based on benthic cover **(B)** and fish abundance **(C)** show each benthic sample replicate using different colors for sites and different shapes for years. The taxonomic compositions of water metagenomes, heterotrophic bacteria, potential coral pathogenic bacteria, and highly abundant bacteria in turf algae are represented by different colors **(D)**. The asterisk in Cyanobacteria represents the bacterial genera *Anabaena, Nostoc*, and *Trichodesmium* (cyanobacteria genera commonly found in turf algae). CFB complex, Cytophaga–Flavobacterium–Bacteroides complex.

**FIGURE 3:**
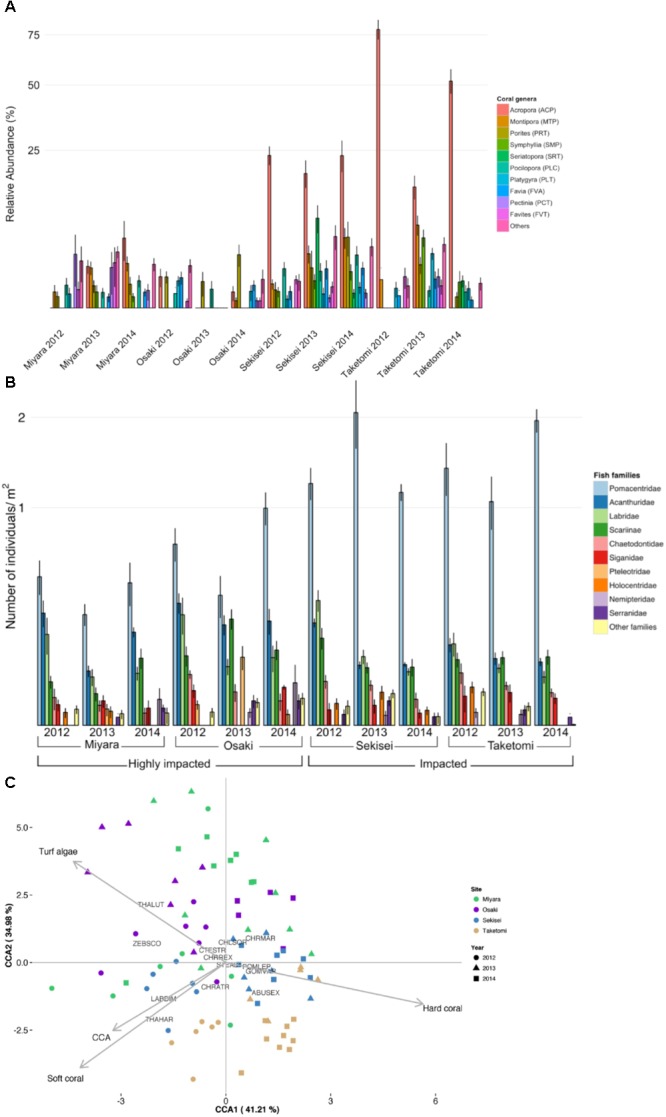
Ishigaki coral reef and fish community composition structure. **(A)** Relative coral cover. **(B)** Relative abundance of fish families. **(C)** Canonical correspondence analysis (CCA) biplot of benthic cover and fish assemblages of Ishigaki coral reefs. Each replicate is represented by dots in different colors (for sites) and different shapes (for years). Species abbreviations are: CTESTR, *Ctenochaetus striatus*; STEALT, *Stegastes altus*; CHLSOR, *Chlorurus sordidus*; POMLEP, *Pomacentrus lepidogenys*; CHRMAR, *Chromis margaritifer*; CHRREX, *Chrysiptera rex*; CHRATR, *Chromis atripectoralis*; ABUSEX, *Abudefduf sexfasciatus*; THALUT, *Thalassoma lutescens*; THAHAR, *Thalassoma hardwicke*; GOMVAR, *Gomphosus varius*; LABDIM, *Labroides dimidiatus*; ZEBSCO, *Zebrasoma scopas*.

Benthic cover differed significantly among sites and sampling years, and the interaction between sites and years was also significant (PERMANOVA, *P* < 0.05) (**Figure 2A** and **Supplementary Table S4**). The nMDS ordination diagram revealed three distinct groups of sites: (i) Miyara, (ii) Osaki, (iii) Taketomi and Sekisei (**Figure 2B**). This grouping supported our first hypothesis that Miyara is the site most affected by land runoff (and nutrient inputs). Turf algae (55.8% ± 2.5; mean ± SE), soft corals (10.8% ± 2.7), and sponges (2.07% ± 0.64) dominated Miyara reef (**Supplementary Figure S2**). Turf algae (85.8% ± 1.2) dominated Osaki reef, the only site where COTS (*Acanthaster planci*) were recorded (**Supplementary Figure S2**). *Acropora* was the dominant coral genus at Sekisei and Taketomi; *Pectinia* and *Porites* were dominant at Miyara and Osaki, respectively (**Figure 3A**). Corals were more abundant at Taketomi (mean 2012–2014: 62.4% ± 2.93) and Sekisei (44.07% ± 2.39) (**Figure 2A**) than at the other reefs, coinciding with the lower wave energy of these two areas. Miyara and Osaki presented a consistently lower coral cover (10.25 ± 1.69 and 4.4 ± 0.74, respectively) (hypothesis 1 confirmed) than the other reefs, and the proportions of corals and turf algae at these two areas were remarkably stable over the study period (**Figure 2A**). Recently, in 2013, dead coral cover increased at Sekisei and Taketomi (17.24% ± 0.9 and 7.75% ± 0.51, respectively) (**Supplementary Figure S3**). The dominance of turf in Miyara reinforces our hypothesis that nutrient inputs from the Miyara River may influence the status of the surrounding reefs. We proceeded to estimate the abundance of fish in an attempt to support hypothesis II. Reduced herbivorous fish assemblages may also contribute to the proliferation of turfs.

### Fish Assemblages

A total of 81 fish species (25 families) were recorded. Fish abundance was overall low, with assemblages dominated by small-sized species (**Figure 2C** and **Supplementary Figures S4, S5**). The total fish abundance was significantly different among sites but not among years (ANOVA, *P* < 0.005; **Supplementary Table S5**). Sekisei and Taketomi had the highest fish abundances (average 0.17 individuals/m^2^), while Osaki and Miyara had the lowest fish abundances (0.12 and 0.06 individuals/m^2^, respectively). Clearly, the overall fish abundance was extremely low in all locations. Pomacentridae (damselfishes) were the most abundant fish at all reefs, with abundances up to 63% of the total number of individuals (**Figure 3B**). At Sekisei and Taketomi, planktivorous damselfishes (e.g., *Pomacentrus lepidogenys, Chromis* spp.) were remarkably more abundant than territorial herbivorous damselfishes (e.g., *Chrysiptera rex, Stegastes* spp.) (**Supplementary Figure S5**). Fish assemblages differed among sites and years, and the interaction between sites and years was also significant (PERMANOVA, *P* < 0.005; **Supplementary Table S6** and **Figures 2C**, 3B). The first two axes of the CCA explained 76.2% of the relationship between habitat characteristics and fish assemblage structure. The main predictor of fish assemblage structure was coral cover, followed by soft coral, turf algae and crustose coralline algae (**Figure 2C**). This pattern of high metagenomic similarity among the studied sites supports hypothesis II and may be related to an ongoing process of microbialization due to the loss of fish biomass and increase in microbial biomass. We proceeded to demonstrate that all studied reefs are under intense hydrodynamic stress that could function as an extra stress factor under these conditions.

### Physical Chemical Water Analysis

The DOC concentration varied between 92 μM at Osaki, 2014, and 233 μM at Miyara, 2013. The highest value of chlorophyll a was observed at Miyara in 2014 (3 μg/L), and the lowest value of chlorophyll a was observed at Sekisei in 2013 (0.2 μg/L). There was no significant difference in DOC among years and sites. Concentrations of total dissolved inorganic nitrogen (TDIN) decreased sharply from 2012 to 2014 at all reefs. The highest values were recorded in 2012 at Miyara (5.4 μM), Osaki (4.3 μM), and Sekisei (3.9 μM), and the lowest values were recorded in 2014 at Osaki (0.6 μM), Sekisei (0.75 μM), Taketomi (0.8 μM), and Miyara (1.2 μM). Over the study period, ammonium decreased significantly (ANOVA *P* < 0.05; **Supplementary Table S9**) at all sites. Nitrate varied significantly only among sites (ANOVA *P* < 0.05; **Supplementary Table S9**), with Miyara presenting the highest concentrations (1.8 μM in 2012) and Osaki presenting the lowest concentrations (0.4 μM in 2014). Nitrite significantly differed among sites but not among years, with Osaki presenting the highest values (0.2 μM in 2013) and Miyara presenting the lowest values (0.2 μM in 2014) (ANOVA, *P* < 0.05; **Supplementary Table S9**). Phosphate concentrations were similar among sites and varied between 0.11 and 0.08 μM (ANOVA, *P* < 0.05; **Supplementary Table S9**). The highest silicate values were observed at Sekisei (1.7 μM in 2013), and the lowest silicate values were observed at Miyara (1.3 μM in 2012).

### Microbial Abundance

The total microbial abundance varied from 1.0 × 10^5^ cells/mL (Taketomi, 2014) to 3.1 × 10^5^ cells/mL (Taketomi, 2012) (**Supplementary Table S1**). Microbial counts were significantly different among sites and years (ANOVA, *P* < 0.05; **Supplementary Table S7**). Vibrio counts were significantly different among sites, and the interaction between sites and years was also significant (ANOVA, *P* < 0.05; **Supplementary Table S8**). The lowest vibrio counts were recorded at Sekisei, 2014 [(24.3 ± 1.0 CFU/ml (*N* = 7)], and Taketomi, 2013 [37.1 ± 0.8 CFU/ml (*N* = 7)], while the highest vibrio counts were observed at Osaki, 2013 [137.1 ± 4.6 CFU/ml (*N* = 7)], and Miyara, 2014 [130.6 ± 3.4 CFU/ml (*N* = 8)] (**Supplementary Table S1** and **Supplementary Figure S6**). These two sites had the highest nutrient values and the lowest coral cover values (**Figure 3A**). The higher abundances of fast-growing vibrio bacteria in these sites is a relevant finding that supports hypothesis II.

### Microbial Community Structure

A total of 19,146,788 high-quality metagenomic reads from 9 seawater samples were generated (**Supplementary Tables S2, S3**). The median values of taxonomically and functionally identified reads were 41.35% (varying from 19.21%, *N* = 437,356, to 60.85%, *N* = 720,291), and 27.89% (13.88%, *N* = 315,932, to 33.86%, *N* = 18,076), respectively (**Supplementary Tables S2, S3**). Most sequences were annotated as *Bacteria* (>87%). *Proteobacteria* reads were the most abundant at all sites (at least 48.53%, *N* = 291,028, at Miyara, 2014), followed by *Cyanobacteria* (at least 7.12%, *N* = 14,993, at Taketomi, 2013) and *Bacteroidetes* (at least 6.05%, *N* = 40,828, at Sekisei, 2013) reads (**Supplementary Figure S7**). Most reads corresponded to heterotrophic bacteria, ranging from 78.1% (*N* = 468,376) at Miyara in 2014 to 92.1% (*N* = 178,943) at Taketomi in 2013 (**Figure 2D**).

**FIGURE 4:**
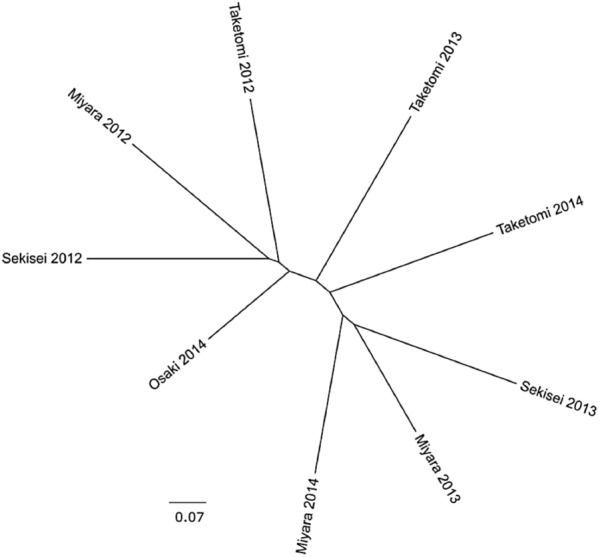
The microbial communities of Ishigaki coral reef seawater have similar genetic compositions. Cladograms representing the fraction of cross-contigs (i.e., shared contigs that contain reads from two or more metagenomes) after cross-assembly.

The percent of reads identified as potential coral pathogens [*Vibrionales, Cytophaga*–*Flavobacterium*–*Bacteroides* complex (CFB complex), *Rickettsiales, Neisseriales*, and *Alteromonadales*] varied from 6.9% (Miyara, 2013) to 18.2% (Sekisei, 2013) (**Figure 1D**). The percent of reads related to highly abundant bacterial groups in turf algae [*Roseobacter, Maribacter, Congregibacter, Dinoroseobacter*, and *Cyanobacteria* (*Anabaena, Nostoc*, and *Trichodesmium*)] varied from 2.55% (Sekisei, 2013) to 9.16% (Sekisei, 2012) (**Figure 2D**). Unlike for the abundance of the fish and benthic assemblages (**Figure 3**), we did not find strong grouping patterns for the metagenomes of the microbial communities based on the taxonomic (class level; **Supplementary Table S10**) and functional annotations (SEED subsystem level 1; **Supplementary Table S11**), dinucleotide composition (**Supplementary Table S12**) and cross-contigs (i.e., shared contigs containing reads from two or more metagenomes) after cross-assembling (**Figure 4**).

## Discussion

The Ishigaki Island reefs have a 10-fold lower abundance of fish and other important herbivores (e.g., sea urchins) than other reef sites (**Table 1**). In addition, the nutrient levels measured in these reefs are higher than the previously reported eutrophication thresholds for Pacific reefs (Bell et al., 2007). Agriculture runoff is the major source of these high nutrient concentrations in the Ishigaki Islands (Tanaka et al., 2011). Nutrification, coupled with a low abundance of herbivores, is a well-known trigger of the transition from coral to algal dominance stages (Hughes, 1994; Birrell et al., 2008). Even under high nutrient concentrations, herbivory can control algal growth and impede algal dominance (Jessen et al., 2013). In Okinawa, an herbivore-exclusion experiment resulted in increased turf algae abundance and prevented coral growth (Tamai and Sakai, 2013), reinforcing the algal dominance mechanism.

**Table 1:** Ishigaki Island reef sites compared with other reefs.

	Ishigaki				Abrolhos		Line Islands	
	Miyara	Osaki	Sekisei	Taketomi	Sebastião Gomes	Parcel dos Abrolhos	Kiritimati	Kingman
Coral cover (ind./m^2^)	10.19	4.03	45.42	63.72	0.33	33.33	21^∗^	71^∗^
Turf algae cover (ind./m^2^)	59.29	92.17	45.55	34.28	93.23	29.94	68^∗^	36^∗^
Fish abundance (ind./m^3^)	0.74	1.35	1.93	1.84	17.2	69.3	4	12
Microbial abund. (cells/ml)	1.40E+05	1.52E+05	1.78E+05	2.02E+05	6.62E+05	4.88E+05	8.4E+5	7.2E+04
Heterotrophs (%)	81	85	83.83	90.01	91	71	50	16
Vibrio (CFU/ml)	71.68	90.34	46.51	50.37	68000	0	940	0
DOC (μM)	166.67	95.84	170.84	164.17	67.4	41.8	51.2	32.3

*Ishigaki fish, benthic, and microbial data are given as the average of the three sampling years for each site. Benthic cover values are shown as percentages; fish abundances are shown as individuals/m^2^; microbial abundances are shown as cells/mL; heterotroph abundances are shown as percentages; Vibrios values are shown as CFU/mL; DOC values are shown as μM. Data from other studies are presented as the minimum and maximum values observed in all studied reefs. Benthic cover and microbial data for Abrolhos were retrieved from (Francini-Filho et al., 2013) and (Bruce et al., 2012), respectively. Data for the Line Islands were retrieved from (Sandin et al., 2008) and (Dinsdale et al., 2008). ^∗^(Sandin et al., 2008) presented coral cover as CCA and stony coral cover together and fleshy algae cover as macro-algae and turf algae cover together.*

### Abundance of Small-Bodied Damselfishes (Pomacentridae)

Overfishing deeply alters the structures of reef fish assemblages via direct (e.g., removal of large predators) and cascading (e.g., increase in prey species) effects. For instance, reefs that are fully open to intense fisheries tend to have a low abundance of large-sized carnivores and herbivores and a higher abundance of small-bodied damselfishes (Pomacentridae) (Edwards et al., 2014). Damselfishes dominated the Ishigaki reef fish communities. However, the proportion of territorial herbivorous species was the highest in Miyara and Osaki, the sites with the lowest coral cover. In Osaki, the site where we recorded the greatest decline in coral cover, the proportions of territorial herbivorous and planktivorous damselfishes also varied significantly, with a marked increase in the abundance of territorial herbivorous species (**Supplementary Figure S5**). Territorial herbivorous damselfish species may positively influence turf algae growth by farming and the aggressive protection of their territories against other herbivorous fish (Arnold et al., 2010; Doropoulos et al., 2013). In addition, the territories of damselfishes may harbor coral pathogenic bacteria (Casey et al., 2014, 2015). Therefore, territorial damselfish may negatively influence coral recovery after mass coral mortality events caused by typhoons and COTS outbreaks.

### Insights on Microbialization

In coral reefs, an increased dominance of fleshy non-calcifying reef builder (e.g., turf and macroalgae) over calcifying reef builder (e.g., corals and coralline algae) organisms is often accompanied by major changes in the microbial community composition and abundance (McDole et al., 2012; Meirelles et al., 2015a; Roach et al., 2017). Nutrification, together with DOC addition from increased benthic algae exudation, further promotes the proliferation of microorganisms (Barott and Rohwer, 2012). Accordingly, we recorded increased abundances of heterotrophic and potentially pathogenic bacteria across the study region, and the DOC concentrations were overall high and comparable to those of other impacted tropical reefs (Dinsdale et al., 2008; Tanaka et al., 2011; Bruce et al., 2012). While DOC may be terrestrially sourced (Tanaka et al., 2011), its high levels and low spatial heterogeneity indicate major autochthonous sourcing. The combined effects of high nutrient concentrations, increased turf algae cover and low fish abundances are pivotal for the establishment of a microbialization process (McDole et al., 2012), as evidenced in our microbial counts and metagenomes, which supports our study hypotheses. The heterotrophic microbial community recorded in Ishigaki included cultured vibrios that have been implicated in coral diseases and mortality, as well as other microbial groups that are typically increased in multifactorial coral diseases (e.g., Bacteroidetes, Fusobacteria, Gammaproteobacteria, Nostocales) (Dinsdale et al., 2008; Bruce et al., 2012; Garcia et al., 2013). In a previous study at the same sampling sites, we found high abundances of globally recognized potential coral-pathogenic vibrio species using a culture-dependent approach (Amin et al., 2016). Indeed, the vibrio counts were significantly higher at Osaki and Miyara, the two reefs with a relatively high turf cover and low fish counts, than at the other reefs. Compared with healthy and impacted reefs from other locations, the benthic community structure of the Ishigaki reefs appears to be in an ongoing coral-to-algal shift in dominance regimes. We observed sites under different coral-turf algae dominance stages, coupled with extremely low fish abundances. In the Line Islands, the highest microbialization levels were observed in the reefs with the lowest fish biomasses (Kelly et al., 2014). Nevertheless, the lowest fish biomass in the Line Island study was at least 10-fold higher than the average fish biomass of the Ishigaki reefs (**Table 1**).

### Extreme Environmental Conditions and Reef Resilience

According to Project Monitoring-Site 1000 reports^1^, coral reefs from Ishigaki have experienced recent coral mass mortality events caused by both coral disease and COT outbreaks and typhoons. From 2007 to 2013, coral cover has generally decreased (to approx. 30%), with only few sites (e.g., Sekisei Lagoon) showing signs of coral cover recovery. Coral transplantation methods have been proposed to restore coral reefs from Okinawa (Omori, 2011); however, the microbialized environment dominated by turf algae and territorial damselfishes coral propagules may not favor coral propagule fixation and growth. In a large data set from more than fifty Central Pacific Islands, no evidence of phase shifts was found; however, fleshy non-calcifying organisms dominated benthic communities of human-populated islands (Smith et al., 2016). According to new definitions of reef health based on reef-building capacity (Smith et al., 2016), Ishigaki coral reefs can be classified as degraded. The new water quality, microbial abundance and diversity evidence provided in the present study reinforces this classification.

Extreme hydrodynamics (e.g., typhoons) may enhance microbialization via different processes, including (i) microbial dispersion and homogenization, as observed in the present study, (ii) loss of coral cover and the opening of benthic habitats for rapid colonization by turfs, and (iii) feedback processes that further alter benthic cover [phase shifts promoting increases in dissolved organic carbon (DOC) and allelopathic compounds produced by turf/algae]. The negative feedback of nutrification and herbivore overfishing has driven several coral reefs to an algae-dominated state (Hoegh-Guldberg et al., 2007). Algae, in turn, are responsible for the production of allelopathic toxic compounds (Nelson et al., 2013), as well as the massive exudation of labile organic matter, which increases microbial abundance and activity (Haas et al., 2011). DOC produced by algae, especially turf algae, promotes the rapid growth of heterotrophic microbial communities and pathogenic bacteria (Smith et al., 2006). In coral-algal interfaces, heterotrophic bacteria may also cause hypoxia, promoting further tissue loss and coral death (Gregg et al., 2013; Jorissen et al., 2016). Overfished reefs that interact with agricultural and urban landscapes tend to lose their resilience and are dominated by heterotrophic microbial communities with high abundances of opportunistic and potential pathogens (Dinsdale et al., 2008; Bruce et al., 2012).

The two reefs with an increased algal dominance (Miyara and Osaki) are under consistently higher wave energy and episodic typhoons than the other reefs. The microbiomes of all the studied sites were relatively similar, reinforcing the possible role of hydrodynamics in the dispersion of microbes across the entire study area. Tropical cyclones have accounted for most of the coral decline recorded in Australia’s Great Barrier Reef (GBR) (De’ath et al., 2012), and the importance of storms may be even higher in the high-latitude reefs of Southern Japan, where the frequency and intensity of cyclones are increasing (Tu and Chou, 2013). A recent study showed that typhoons can cause shifts in coral morphology dominance (i.e., from foliose to bushy) in the upper mesophotic coral reefs from Okinawa (White et al., 2017). Storms and typhoons have been reported to be associated with increases and shifts in phytoplankton compositions and to influence the calcification of corals in Ishigaki reefs (Blanco et al., 2008; Sowa et al., 2014). In the Caribbean, strong wave swells have increased diseases and snail predation in coral (Bright et al., 2016). In addition, COTS outbreaks seem to play a major role in reef resilience across the Pacific Ocean, including Japan (Nishihira and Yamazato, 1974; Sano, 1987) and the GBR, where COTS is the second most important stressor to coral communities (De’ath et al., 2012). During our study, we recorded a COTS outbreak in Osaki, and the coral cover of Miyara was drastically reduced due an outbreak in 2011 (T. Sawabe unpub. obs.). After a COTS outbreak, coral cover recovery may take years. For instance, coral cover recovery in Guam, Micronesia, was much faster (from 0.9 to 65% in 3 years) (Colgan, 1987) than that reported in the present study. Remarkably, COTS outbreaks are related to the increasing survival of plankton-feeding larvae in nutrient-enriched flood waters (Fabricius et al., 2010). After mass coral mortality events, coral community compositions can change. In our study, we found that, after COTS outbreaks, massive colonies (*Porites* and *Pectinia*) were dominant over branching colonies (*Acropora* and *Montipora*). Similar results were obtained in another study at the Ishigaki Island reefs (Harii et al., 2014). Increasing coverage of massive coral colonies rather branching coral colonies is associated with a loss in reef structural complexity and a decrease, based on the number of scales, coral recovery by reducing both the delivery of coral larvae to the substratum and larval settlement (Hata et al., 2017).

## Conclusion

Our analysis has provided new insights into the microbial diversity of Ishigaki reefs. Here, we suggest that microbialization may be an important mechanism (via the DDAM model) influencing the coral community and can be used as an early warning signal for coral reef phase shifts in the Ishigaki reefs. The high latitude and high hydrodynamics in the Ishigaki coral reefs mean these reefs suffer periodic acute declines in coral cover due to, e.g., typhoons. The system’s capacity to return to coral-dominated states after periodic typhoons is being increasingly compromised, possibly by an ongoing microbialization process. Hydrodynamics may play fundamental roles in coral reef microbialization. We suggest the inclusion of hydrodynamic analyses for future studies regarding coral reef microbialization. The proliferation of rapidly growing heterotrophic microbes (e.g., vibrios and other potential coral pathogens) demonstrated in the present study hints to important features of these Ishigaki reefs. Overfishing has nearly extinguished fish from these reefs, while turf and nutrification have promoted microbialization. Increased DOC from turf algae and runoff contribute to benthic assemblages under different coral-algal dominance stages. These stressors interact in positive feedback loops that enhance microbialization and reduce reef resilience.

## Materials and Methods

### Study Area

Located in the southwestern part of the Ryukyu Islands, Ishigaki Island (approx. area 229 km^2^ with approx. 49,075 habitants) is surrounded by fringing reefs exposed to high levels of anthropogenic and terrestrial influences (e.g., river, ground, and beach water) (Kawahata et al., 2000). Miyara River, the longest river of the island, has a catchment area of 35.4 km^2^ and total length of approx. 12 km; considerable amounts of red soil and nutrient from the fields, which are emitted by animal wastes and chemical fertilizers from agricultural activities, outflow from this river (Banzai et al., 2003; Banzai and Nakamura, 2006; Abe, 2010).

We selected four sites exposed to different local stressors in Ishigaki Island, Okinawa, Japan (**Supplementary Figure S1** and **Supplementary Table S1**). We classified the reef sites as “impacted” based on previous coral declines due to mass mortality and/or COTS outbreaks (Program Monitoring Sites 1000 2007–2013 reports^2^) and “highly impacted” based on previous coral cover data and previous and ongoing COTS outbreaks (T. Sawabe unpub. obs.). The Taketomi (24°20.5260′N, 124°05.6443′E) and Sekisei reefs (24°21.7557′N, 124°02.7190′E, inside Iriomote Ishigaki National Park) were classified as “impacted,” and the Miyara (24°20.5489′N, 124°13.0408′E) and Osaki reefs (24°25.4171′N, 124°04.4956′E) were classified as “highly impacted.” Sampling occurred after the rainy season and before the typhoon season, in June 2012, 2013, and 2014. No specific permissions were required. All sampling efforts were conducted at the reef front.

### Benthic Cover

Benthic cover was estimated following previously described procedures (Francini-Filho and de Moura, 2008; Francini-Filho et al., 2013). At each site, 15 photoquadrats with 0.7 m^2^ were randomly placed in similar reef sections of approx. 100 m^2^. In the subsequent sampling years, samples were obtained at the same positions, using GPS markings. Percent cover was estimated using Coral Point Count with Excel Extension software (CPCe) (Kohler and Gill, 2006), with 100 randomly distributed points per photoquadrat. Organisms below each point were identified and grouped in the following major benthic functional categories (Steneck and Dethier, 1994): turf algae, fleshy algae, sponges, echinoderms, tunicates, mollusks, sand, and corals (at the genus level). The health of each coral colony was classified into one of three categories: healthy, diseased (e.g., signs of tissue necrosis and bleaching), and recently dead (exposed skeleton).

### Fish Assemblages

Fish assemblages were assessed from video records by SCUBA divers using a Sony HDR-XR550 V camera. Samples were obtained at the same sites where water samples and benthic cover estimates were obtained, at approx. 5 m depth. Recordings (*N* = 7 per site in each year) were made under slow movement near the bottom (approx. 1 m). Fish counts in the video footage were standardized by space and time (10′ video stretches, *N* = 83). Trophic guild assignments were based on literature data (Randall, 1967). The results are presented as number of individuals and relative abundance.

### Physical, Chemical, and Biological Inventories

Divers collected water samples at the benthic boundary layer (up to 10 cm from the bottom) using a Beckson Thirsty-Mate^®^ hand pump and clean 10 L water gallons. Temperature, Dissolved Oxygen (DO) and salinity were measured *in situ* using a U-50 Multiparameter Water Quality Checker (Horiba, Tokyo). Chlorophyll *a*, DOC, inorganic nutrients and microbial abundances were determined following previously described methods (Andrade et al., 2003; Grasshoff et al., 2009; Rezende et al., 2010). Chlorophyll *a* samples were collected using negative pressure filtration with 1–2 L of water. Filters (GF/C, Whatman^®^, GE Healthcare) were extracted in dimethylformamide at -20°C and analyzed by a Turner Design fluorometer. For the DOC analysis, 50 mL of seawater filtered using a calcinated and weighted Whatman GF/F glass microfibre filter was collected in a HCl-washed amber bottle and fixed with 100 μL of 85% phosphoric acid. Fixed samples were tightly sealed and refrigerated at 4°C to avoid microbial degradation until DOC quantification procedures could be conducted (Sharp et al., 2002; Shimada et al., 2010). The detection limits for DOC and nutrient analyses were 0.83 μmol/L (0.01 mg C/L) and 0.01 μmol/L, respectively, based on three times the standard deviation of the lowest concentration of the samples. The analytical precision (coefficient of variation, CV) was 2–3% by replicate measurements of each sample. For inorganic nutrient analyses, 10 mL of water was frozen and analyzed in the laboratory using an auto-analyser (Bran+Luebbe, Autoanalyzer II) (Shimada et al., 2010). Microbial abundance was determined by DAPI staining (Kepner and Pratt, 1994) under an Axioo Photo epifluorescence microscope (Zeiss, Germany) (Porter and Feig, 1980; Martinussen and Thingstad, 1991). Colony forming units (CFUs) of vibrios were estimated using 0.2 mL aliquots of seawater plated on TCBS selective medium (10 replicates) incubated at room temperature. Counts were performed up to 48 h after plating.

### Coral Reef Seawater Metagenomic DNA Extraction

Water samples from each site were collected near the bottom (<10 cm) and filtered in four Sterivex (0.22 μm) filters. Four independent replicates of seawater were prefiltered through nets of 100 μm and 20 μm by gravity. Two liters of water per replicate were filtered. Microbial cells retained in the filter received SET buffer and were stored at 30°C until DNA extraction. DNA extraction was performed using modified column purification protocol (Nucleospin Tissue, Macherey-Nagel, Dueren, Germany), as previously described (Bruce et al., 2012). A pool of DNA extracted from the filters was used for sequencing. Quality control was performed with Nanodrop absorbance and quantification by using the 2100 Bioanalyzer and Qubit High Sensitivity DNA Kit (Agilent, Santa Clara, CA, United States).

### Metagenome Sequencing and Sequence Analysis

Metagenomic DNA samples were sequenced by Illumina MiSeq (paired-end sequencing, 2 × 300 base pairs). Metagenomic libraries were prepared using the Nextera and Nextera XT Sample Preparation Kits (Illumina, San Diego, CA, United States). The size distribution of reads was accessed with the 2100 Bioanalyzer and the High Sensitivity DNA Kit (Agilent, Santa Clara, CA, United States). Quantification of libraries was performed with the 7500 Real Time PCR system (Applied Biosystems, Foster City, CA, United States) and the KAPA Library Quantification Kit (Kapa Biosystems, Wilmington, MA, United States).

### Quality Control and Metagenomic Data Analysis

Low quality (quality score <30) and duplicate sequences were removed using PRINSEQ (Schmieder and Edwards, 2011). Metagenomic sequence annotation was conducted using MG-RAST v3.5 (Meyer et al., 2008) with a maximum *e*-value cut-off of 1 × 10^-5^. Taxonomic and functional annotation was performed using the SEED database. To standardize the annotated metagenome sizes, we presented data as relative abundances (number of sequences of a given taxa or subsystem divided by the total number of identified sequences of the metagenome). Reads from all nine metagenomes were combined into a single fasta file and were cross-assembled using Mira (Chevreux et al., 2004). The results were visualized using the metagenome cross-assembly tool crAss (Dutilh et al., 2012). Briefly, crAss calculates a distance matrix between all pairs of metagenomes and corrects for sample size using the SHOT formula, which has previously been used to correct for genome size when calculating phylogenetic distances (Korbel et al., 2002; Dutilh et al., 2004, 2007). This distance matrix was converted into a cladogram using BioNJ (Gascuel, 1997) and was visualized using FigTree^3^. All data are available at BaMBa (Meirelles et al., 2015b) (pmeirelles.19), and the metagenomes are also available in MG-RAST servers. The unique metagenome identifiers are listed in **Supplementary Tables S2, S3**.

### Statistical Analysis

Analyses were performed with R (R Development Core Team, 2011), except where indicated. Abundance and multivariate figures were plotted with the packages ggplot2 and reshape. Non-metric multidimensional scaling (nMDS) ordination was used to summarize spatial and temporal similarities (Bray-Curtis) of benthic, fish, and microbial community structures using the metaMDS in the vegan package (Oksanen et al., 2005). To test if benthic, fish, and microbial assemblages (both taxonomically and functionally) differed among sampling sites and years, permutational multivariate analysis of variance (PERMANOVA) was performed using the function adonis (Oksanen et al., 2005) (Bray-Curtis distances and 999 permutations). To investigate the relationship between: (i) fish and benthic assemblages; (ii) microbial and benthic assemblages, and (ii) microbial assemblages and environmental variables (e.g., nutrients, dissolved oxygen, depth), we performed a canonical correspondence analysis (CCA) using the cca function (Oksanen et al., 2005). Only the most abundant species (>90% of individuals) were used in the CCA. The Monte Carlo permutation test was used to test for the statistical significance (*P* < 0.05) of the contribution of each variable in the CCA axes. Bacterial diversity indices [Shannon entropy and Shannon evenness (i.e., Hill’s Ratio)] and richness were determined with data at the species level (Oksanen et al., 2005). To assess the genetic similarity of seawater microbiomes among reef sites and years, the dinucleotide compositions of seawater metagenomes were compared. Frequency tabulation of sequence data was performed according to (Willner et al., 2009) with a homemade Python script. A principal component analysis (PCA) was performed using the rda function (Oksanen et al., 2005) in order to visualize sample grouping. To test the hypothesis that the seawater microbiomes are genetic similar, the dinucleotide composition was analyzed among sites and years. PERMANOVA was performed using the adonis function (Oksanen et al., 2005). For all analysis *P*-values <0.05 were considered statistically significant. For multivariate analysis, percentages data were transformed to arcsin (√x). The results are presented as mean ± standard error.

### Numerical Modeling

To assess the extreme wave conditions around the area of interest, simulated typhoon-generated waves were propagated nearshore with the Delft 3D (Deltares) numerical model. Simulations http://tree.bio.ed.ac.uk/software/figtree/

included offshore wave information extracted from the global wave generation model WaveWatch III (NCEP/NOAA) and typical wind conditions observed during typhoons (e.g., the 2013 SOULIK and the 2014 NEOGURI typhoons). The model domain covered the continental shelf of Ishigaki, Iriomote and adjacent islands, with a grid of 120 by 80 km, keeping the sites of interest well inside the domain. Bathymetry was based on the ETOPO1 Global Relief Model (NOAA). After running the model for the defined conditions, wave data were extracted close to the sites of interest. Based on wave characteristics, wave power was estimated in order to assess its relative impact among sites.

## Author Contributions

PM, TS, CT, and FT conceived and designed the experiments. PM, AS, LO, LL, IK, SM, TS, and FT performed the experiments. PM, RF-F, IK, CT, TS, and FT contributed reagents, materials, and analysis tools. All authors analyzed the data. All authors wrote the paper.

## Conflict of Interest Statement

The authors declare that the research was conducted in the absence of any commercial or financial relationships that could be construed as a potential conflict of interest.

## Footnotes

1 http://www.biodic.go.jp/moni1000/findings/reports/index.html#2464

2 http://www.biodic.go.jp/moni1000/

## Supplementary Material

FIGURE S1 Study sites in Ishigaki Island, Okinawa, Japan. The maps were generated using Qgis software [1].

FIGURE S2 Other benthic categories (complement of manuscript **Figure 2A**). Values are Mean ± Standard error (*N* = 15).

FIGURE S3 Recent coral mortality and disease. Values are Mean ± Standard error (*N* = 15).

FIGURE S4 Total fish abundance. Values are Mean ± Standard error (*N* = 7).

FIGURE S5 Damselfishes (Pomacentridae) trophic guild composition. Values are Mean ± Standard error (*N* = 15).

FIGURE S6 Vibrio counts. Values are Mean ± Standard error (*N* = 3) of colony forming unities.

FIGURE S7 Major microbial phyla. The microbial composition was similar among the reef sites based on bacterial phyla composition **(A)** and other taxonomic and functional levels.

TABLE S1 Information on the sampling sites and chemical and biological inventories of seawater samples.

TABLE S2 General features of the metagenomes.

TABLE S3 Metagenomes taxonomic annotation at Domain level and bacterial family diversity.

TABLE S4 Adonis (Permanova) results of benthic cover based on Bray-Curtis distances with 999 permutations. MS, mean sum of squares; SS, sum of squares.

TABLE S5 ANOVA results of total fish abundance. DF, degrees of freedom; SS, sum of squares; MS, mean sum of squares.

TABLE S6 Adonis (Permanova) results of fish community abundance based on Bray-Curtis distances with 999 permutations. DF, degrees of freedom; SS, sum of squares; MS, mean sum of squares.

TABLE S7 ANOVA results of total bacterial abundance. DF, degrees of freedom; SS, sum of squares; MS, mean sum of squares.

TABLE S8 ANOVA results of Vibrio counts. DF, degrees of freedom; SS, sum of squares; MS, mean sum of squares.

TABLE S9 ANOVA results of nutrients and chlorophyll a concentrations. DF, degrees of freedom; SS, sum of squares; MS, mean sum of squares.

TABLE S10 Adonis (Permanova) results of metagenomic class composition (arcsin transformed) abundance based on Bray-Curtis distances with 999 permutations. MS, mean sum of squares; SS, sum of squares.

TABLE S11 Adonis (Permanova) results of metagenomic level 1 subsystem composition (arcsin transformed) abundance based on Bray-Curtis distances with 999 permutations. MS, mean sum of squares; SS, sum of squares.

TABLE S12 Adonis (Permanova) results of metagenomic dinucleotides composition abundance based on Bray-Curtis distances with 999 permutations. MS, mean sum of squares; SS, sum of squares.

TABLE S13 Wave characteristics extracted based on typhoon wave conditions. Hs: significant wave height (m); Dir: direction in degrees North; P: wave power (W/m).
