# Supplementary figures and images for: Metagenomics of Coral Reefs Under Phase Shift and High Hydrodynamics
Source: Front Microbiol. 2018 Oct 4;9:2203. doi: 10.3389/fmicb.2018.02203 (PMC6180206; doi:10.3389/fmicb.2018.02203)

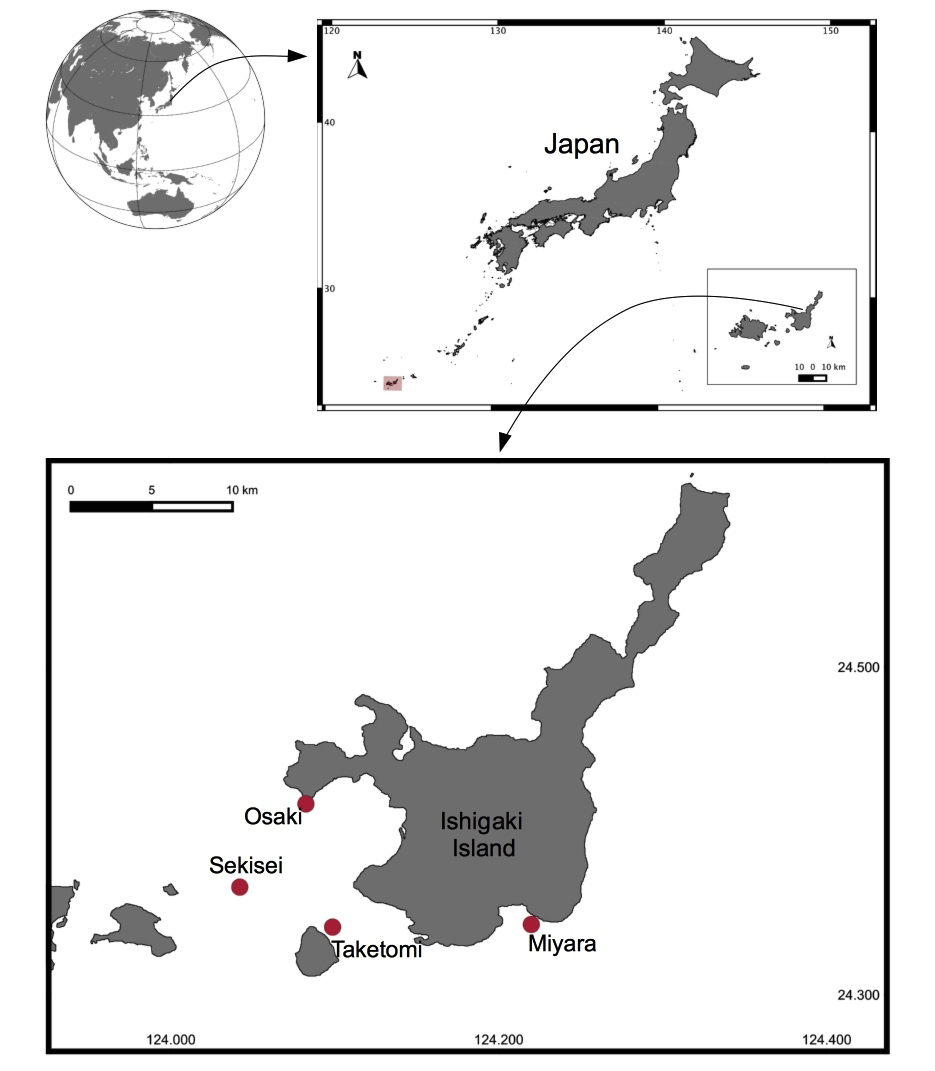

Supplement: FIGURE S1 — Study sites in Ishigaki Island, Okinawa, Japan. The maps were generated using Qgis software [1]. [file Image_1.TIF]

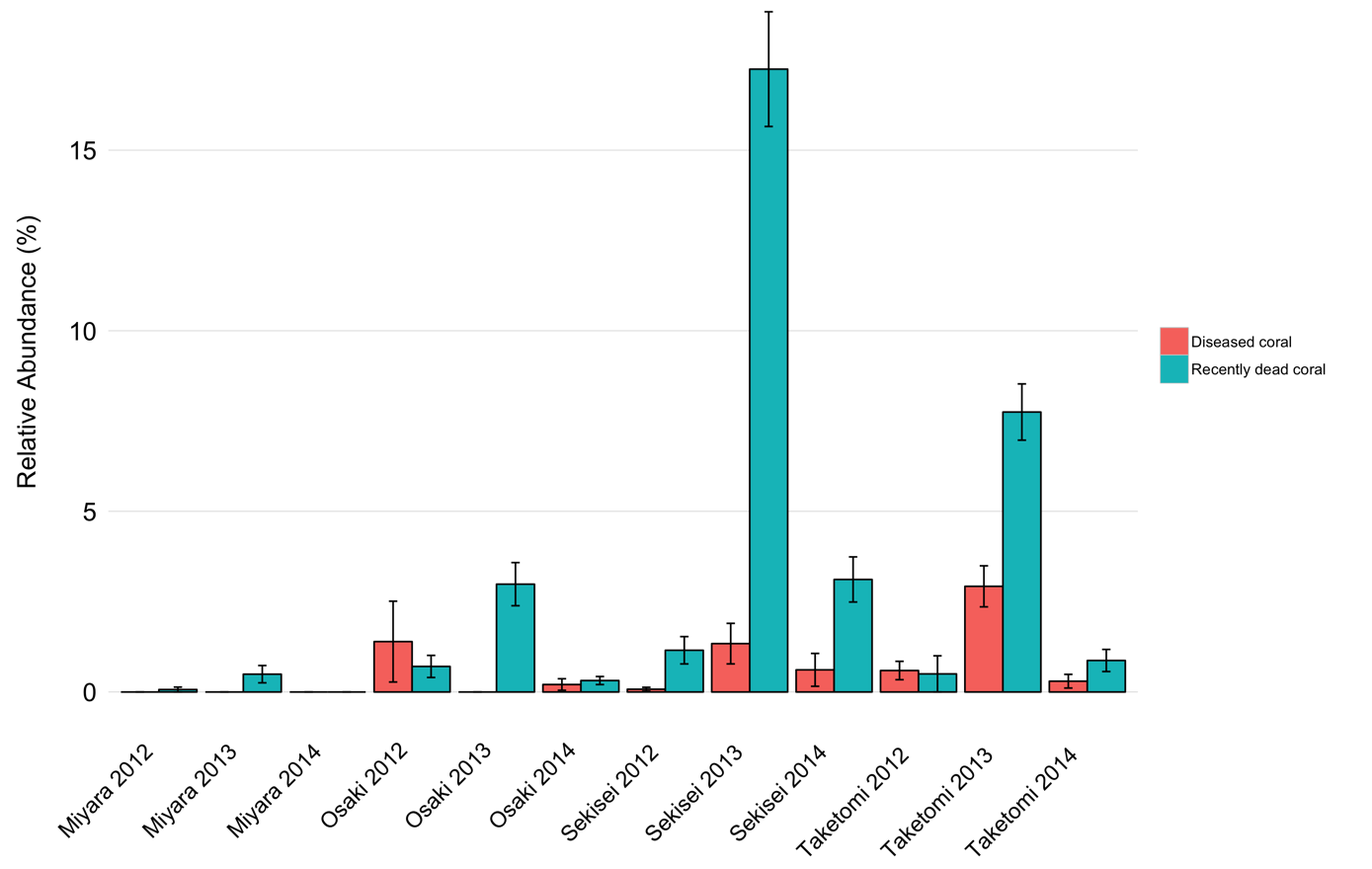

Supplement: FIGURE S3 — Recent coral mortality and disease. Values are Mean ± Standard error (N = 15). [file Image_3.TIF]

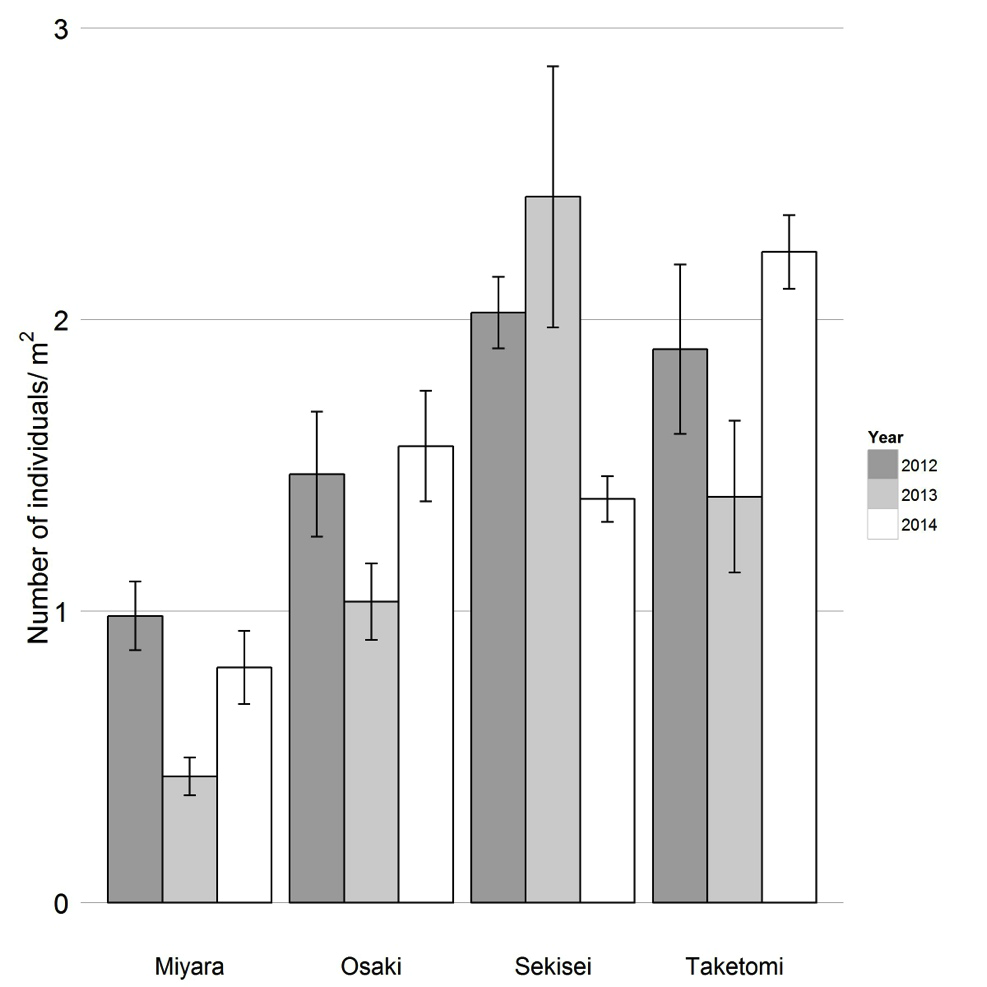

Supplement: FIGURE S4 — Total fish abundance. Values are Mean ± Standard error (N = 7). [file Image_4.TIF]

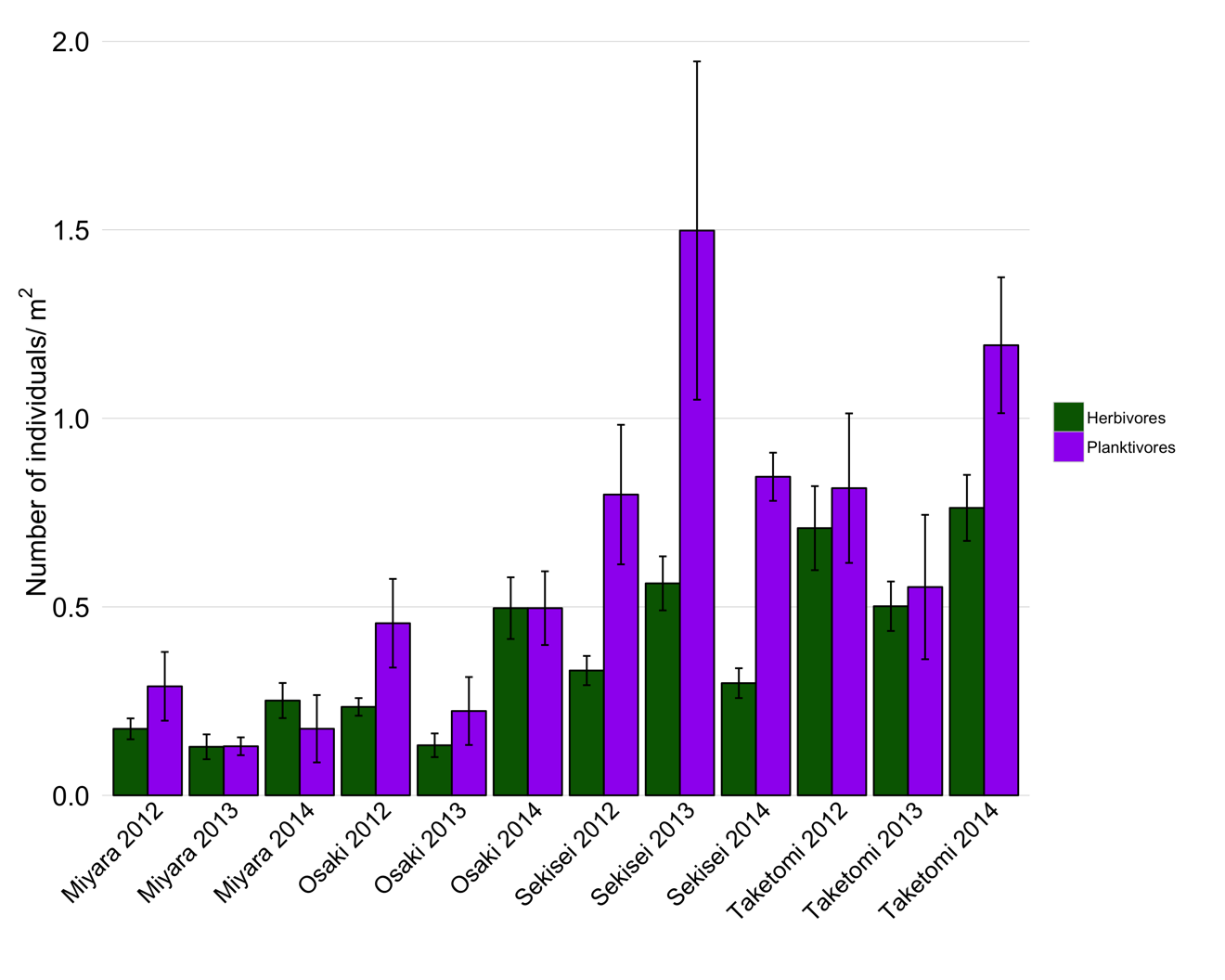

Supplement: FIGURE S5 — Damselfishes (Pomacentridae) trophic guild composition. Values are Mean ± Standard error (N = 15). [file Image_5.TIF]

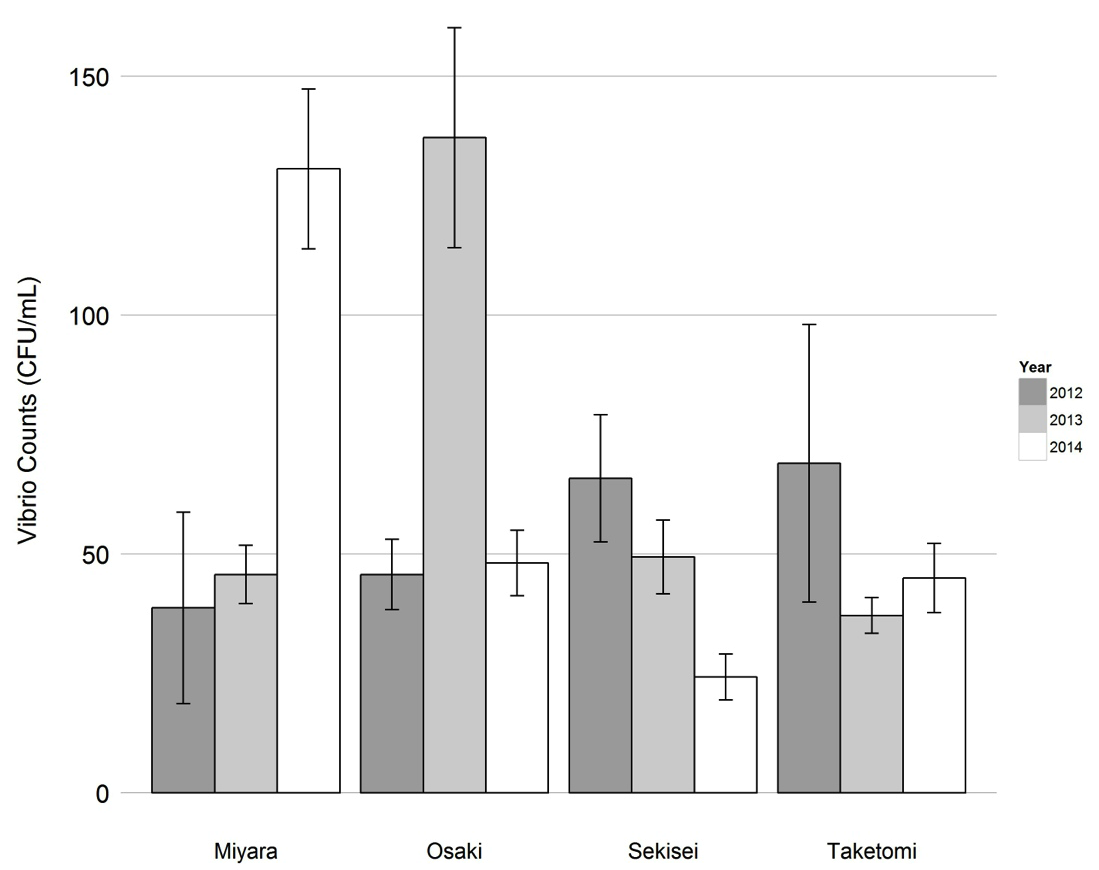

Supplement: FIGURE S6 — Vibrio counts. Values are Mean ± Standard error (N = 3) of colony forming unities. [file Image_6.TIF]
