# Supplementary material for: Metagenomics of Coral Reefs Under Phase Shift and High Hydrodynamics
Source: Front Microbiol. 2018 Oct 4;9:2203. doi: 10.3389/fmicb.2018.02203 (PMC6180206; doi:10.3389/fmicb.2018.02203)
Supplement: TABLE S1 — Information on the sampling sites and chemical and biological inventories of seawater samples. [file Table_S1.doc]

Supplementary Table 1 –Information on the sampling sites and chemical and biological inventories of seawater samples.

| **Site** | | **Miyara** | | | | **Osaki** | | | | **Sekisei** | | | | **Taketomi** | | |
| --- | --- | --- | --- | --- | --- | --- | --- | --- | --- | --- | --- | --- | --- | --- | --- | --- |
| **Year** | 2012 | | 2013 | 2014 | 2012 | | 2013 | 2014 | 2012 | | 2013 | 2014 | 2012 | | 2013 | 2014 |
| **Sampling date** | 27/06/2012 | | 09/06/2013 | 30/06/2014 | 28/06/2012 | | 10/07/2013 | 30/06/2014 | 26/06/2012 | | 10/07/2013 | 02/07/2014 | 26/06/2012 | | 09/07/2013 | 01/07/2014 |
| **Latitude** | 24°20.5489' N | | 24°20.5260' N | 24°20.6440' N | 24°25.4171' N | | 24°25.4171' N | 24°23.208' N | 24°21.7557' N | | 24°21.7557' N | 24°21.6805' N | 24°20.5260' N | | 24°20.5489' N | 24°20.3018' N |
| **Longitude** | 124°13.0408' E | | 124°05.6443' E | 124°12.7506' E | 124°04.4956' E | | 124°04.4956' E | 123°55.681' E | 124°02.7190' E | | 124°02.7190' E | 124°02.8600' E | 124°05.6443' E | | 124°05.0408' E | 124°05.9410' E |
| **Depth (m)** | 4 | | 4 | 4 | 4 | | 4 | 4 | 3 | | 3 | 3 | 4 | | 4 | 4 |
| **DO (mg/L)** | 6.6 | | 5.7 | 8.56 | 6.8 | | 4.2 | 5.5 | 8.1 | | 4.8 | 4.92 | 7 | | 5.5 | 7.44 |
| **Salinity** | 35.0 | | 35.0 | 33.1 | 35.0 | | 34.0 | 32.7 | 35.0 | | 34.0 | 34.1 | 35.0 | | 35.0 | 34.3 |
| **Temperature (°C)** | 27.5 | | 30 | 29.99 | 28.1 | | 30.1 | 30.6 | 28.1 | | 29.9 | 30.48 | 27.7 | | 29.9 | 30.04 |
| **Bacterial counts (cells/mL)** | 1.41E+05 ± 1.21E+04(N= 10) | | 1.11E+05 ± 6.69E+03 (N= 2) | 1.68E+05± 3.20E+03 (N= 2) | 1.58E+05 ±2.11E+04 (N= 9) | | 1.56E+05 ± 4.46E+03(N= 2) | 1.43E+05± 1.91E+03 (N= 2) | 2.50E+05 ± 3.22E+04 (N= 6) | | 1.55E+05 ± 2.07E+04 (N= 2) | 1.30E+05± 1.38E+04 (N= 2) | 3.07E+05 ± 1.37E+05 (N= 5) | | 1.99E+05 ± 2.12E+04 (N= 2) | 1.00E+05± 3.91E+03 (N= 2) |
| **Vibrio counts (CFU/mL)** | 38.75 ± 20.04 (N= 4) | | 45.7 ± 1.22  (N= 7) | 130.6 ± 3.35 (N= 8) | 48.33 ± 6.82 (N= 7) | | 137.14 ± 4.60 (N= 7) | 85.56 ± 7.58 (N= 9) | 65.83 ± 13.32 (N= 6) | | 49.4 ± 1.54  (N= 8) | 24.3 ± 0.96  (N= 7) | 69 ± 29.04  (N= 5) | | 37.1 ± 0.75  (N= 7) | 45 ± 1.45  (N= 7) |
| **Chlorophyll a (ug/L)** | 1.56 ± 0.67  (N= 3) | | 0.30 | 2.97 ± 1.79  (N= 2) | 0.74 ± 0.13 (N=3) | | 0.32 | 1.38 ± 0.30  (N= 2) | 2.60 ± 0.67  (N= 4) | | 0.20 | 1.02 ± 0.48  (N= 2) | 2.65 ± 0.27  (N= 2) | | 0.61 | 1.42 ± 0.54  (N= 2) |
| **DOC (μM)** | - | | 233.33 | 100.00 | - | | 100.00 | 91.67 | - | | 216.67 | 125.00 | - | | 158.33 | 150.00 |
| **POC (μM)** | - | | 0.059 | 0.38 | - | | 0.045 | 0.239 | - | | 0.042 | 0.12 | - | | 0.086 | 0.92 |
| **PO_4_ (μM)** | 0.11 ± 0.01 (N= 4) | | 0.08 ± 0.01 (N= 2) | 0.11 ± 0.00 (N= 2) | 0.10 ± 0.01 (N= 4) | | 0.08 ± 0.06 (N= 2) | 0.19 ± 0.08 (N= 2) | 0.11 ± 0.01 (N= 4) | | 0.08 ± 0.01 (N= 2) | 0.09 ± 0.01 (N= 2) | 0.10 ± 0.01 (N= 5) | | 0.11 ± 0.01 (N= 2) | 0.09 ± 0.00 (N= 2) |
| **NO_2_ (μM)** | 0.21 ± 0.02 (N= 4) | | 0.19 ± 0.07 (N= 2) | 0.11 ± 0.01 (N= 2) | 0.18 ± 0.02 (N= 4) | | 0.22 ± 0.08 (N= 2) | 0.11 ± 0.00 (N= 2) | 0.18 ± 0.02 (N= 4) | | 0.13 ± 0.00 (N= 2) | 0.13 ± 0.00 (N= 2) | 0.16 ± 0.02 (N= 5) | | 0.17 ± 0.01 (N= 2) | 0.13 ± 0.01 (N= 2) |
| **NO_3_ (μM)** | 1.83 ± 0.83 (N= 4) | | 0.91 ± 0.14 (N= 2) | 1.04 ± 0.09 (N= 2) | 0.42 ± 0.07 (N= 4) | | 0.48 ± 0.09 (N= 2) | 0.40 ± 0.04 (N= 2) | 0.63 ± 0.04 (N= 4) | | 0.50 ± 0.07 (N= 2) | 0.62 ± 0.07 (N= 2) | 0.55 ± 0.06 (N= 5) | | 1.05 ± 0.04 (N= 2) | 0.66 ± 0.06 (N= 2) |
| **NH_4_ (μM)** | 3.38 ± 0.74 (N= 4) | | 1.25 ± 1.25 (N= 2) | 0.08 ± 0.01 (N= 2) | 3.66 ± 0.81 (N= 4) | | 2.74 ± 1.97 (N= 2) | 0.10 ± 0.00 (N= 2) | 3.12 ± 0.74 (N= 4) | | 1.44 ± 0.58 (N= 2) | 0.00 ± 0.00 (N= 2) | 1.51 ± 0.73 (N= 5) | | 2.22 ± 0.99 (N= 2) | 0.01 ± 0.01 (N= 2) |
| **SiO_2_ (μM)** | 1.27 ± 0.02 (N= 4) | | 1.69 ± 0.05 (N= 2) | 1.41 ± 0.03 (N= 2) | 1.39 ± 0.04 (N= 4) | | 1.56 ± 0.04 (N= 2) | 1.30 ± 0.00 (N= 2) | 1.23 ± 0.02 (N= 4) | | 1.71 ± 0.03 (N= 2) | 1.30 ± 0.03 (N= 2) | 1.28 ± 0.02 (N= 5) | | 1.70 ± 0.12 (N= 2) | 1.56 ± 0.01 (N= 2) |
