# Supplementary material for: Metagenomics of Coral Reefs Under Phase Shift and High Hydrodynamics
Source: Front Microbiol. 2018 Oct 4;9:2203. doi: 10.3389/fmicb.2018.02203 (PMC6180206; doi:10.3389/fmicb.2018.02203)
Supplement: TABLE S2 — General features of the metagenomes. [file Table_S2.doc]

Supplementary Table 2 – General features of the metagenomes.

| **MG-RAST ID** | **Site** | **Year** | **Total of Sequences (n)** | **Sequence Length (Mean ± SD)** | **Mean GC (%)** | **Sequences assigned to taxonomy (%)** | **Sequences assigned to functional (%)** |
| --- | --- | --- | --- | --- | --- | --- | --- |
| 4620714.3 | Taketomi | 2012 | 1394872 | 249 ± 56 | 45 ± 11 | 638557 (45.78%) | 468657 (33.60%) |
| 4619069.3 | Taketomi | 2013 | 526272 | 344 ± 119 | 36 ± 6 | 222634 (42.30%) | 152142 (28.91%) |
| 4618925.3 | Taketomi | 2014 | 66203 | 272 ± 113 | 37 ± 6 | 25775 (38.93%) | 22115 (33.40%) |
| 4620713.3 | Sekisei | 2012 | 2276142 | 189 ± 80 | 40 ± 8 | 437356 (19.21%) | 315932 (13.88%) |
| 4618924.3 | Sekisei | 2013 | 1183749 | 353 ± 109 | 35 ± 6 | 720291 (60.85%) | 395398 (33.40%) |
| 4620712.3 | Miyara | 2012 | 53383 | 224 ± 70 | 43 ± 11 | 20110 (37.67%) | 18076 (33.86%) |
| 4618923.3 | Miyara | 2013 | 2257717 | 268 ± 104 | 35 ± 6 | 1094948 (48.50%) | 517145 (22.91%) |
| 4618922.3 | Miyara | 2014 | 1359313 | 316 ± 98 | 36 ± 6 | 658291 (48.43%) | 367823 (27.06%) |
| 4620881.3 | Osaki | 2014 | 10029137 | 183 ± 17 | 44 ± 12 | 3052938 (30.44%) | 2401617 (23.95%) |
