# Supplementary material for: Metagenomics of Coral Reefs Under Phase Shift and High Hydrodynamics
Source: Front Microbiol. 2018 Oct 4;9:2203. doi: 10.3389/fmicb.2018.02203 (PMC6180206; doi:10.3389/fmicb.2018.02203)
Supplement: TABLE S3 — Metagenomes taxonomic annotation at Domain level and bacterial family diversity. [file Table_S3.doc]

Supplementary Table 3 – Metagenomes taxonomic annotation at Domain level and bacterial family diversity.

| metagenome | Reef | Year | Bacteria (%) | Eukaryota (%) | Viruses (%) | Archaea (%) | other sequences (%) | Unassigned (%) | Bacterial species richness | Shannon entropy | Shannon Evenness |
| --- | --- | --- | --- | --- | --- | --- | --- | --- | --- | --- | --- |
| 4620714.3 | Taketomi | 2012 | 585562 (91.70%) | 5390 (0.84%) | 7416 (1.16%) | 3613 (0.57%) | 0 (0%) | 36574 (5.73%) | 637 | 4.73142 | 0.05513134 |
| 4619069.3 | Taketomi | 2013 | 194241 (87.25%) | 3217 (1.44%) | 9060 (4.07%) | 1512 (0.68%) | 0 (0%) | 14604 (6.56%) | 608 | 4.000199 | 0.01369186 |
| 4618925.3 | Taketomi | 2014 | 23523 (91.26%) | 237 (0.92%) | 385 (1.49%) | 155 (0.6%) | 0 (0%) | 1475 (5.72%) | 550 | 4.278676 | 0.03346217 |
| 4620713.3 | Sekisei | 2012 | 396013 (90.55%) | 8849 (2.02%) | 4464 (1.02%) | 1959 (0.45%) | 1 (0.0%) | 26070 (5.96%) | 617 | 4.630309 | 0.0483726 |
| 4618924.3 | Sekisei | 2013 | 674608 (93.66%) | 3736 (0.52%) | 15439 (2.14%) | 3649 (0.51%) | 67 (0.01%) | 22792 (3.16%) | 632 | 3.986469 | 0.02052016 |
| 4620712.3 | Miyara | 2012 | 18656 (92.77%) | 182 (0.91%) | 199 (0.99%) | 105 (0.52%) | 2 (0.01%) | 966 (4.8%) | 540 | 4.533021 | 0.04633862 |
| 4618923.3 | Miyara | 2013 | 997022 (91.06%) | 10007 (0.91%) | 28032 (2.56%) | 6746 (0.62%) | 11 (0.00%) | 53130 (4.85%) | 636 | 4.131394 | 0.0171514 |
| 4618922.3 | Miyara | 2014 | 599656 (91.09%) | 7083 (1.08%) | 12240 (1.86%) | 4471 (0.68%) | 8 (0.00%) | 34833 (5.29%) | 635 | 4.114899 | 0.01873885 |
| 4620881.3 | Osaki | 2014 | 2771871 (90.79%) | 39611 (1.3%) | 68401 (2.24%) | 19467 (0.64%) | 11 (0.00%) | 153577 (5.03%) | 661 | 4.646947 | 0.03264833 |
