# Supplementary material for: Metagenomics of Coral Reefs Under Phase Shift and High Hydrodynamics
Source: Front Microbiol. 2018 Oct 4;9:2203. doi: 10.3389/fmicb.2018.02203 (PMC6180206; doi:10.3389/fmicb.2018.02203)
Supplement: TABLE S4 — Adonis (Permanova) results of benthic cover based on Bray-Curtis distances with 999 permutations. MS, mean sum of squares; SS, sum of squares. [file Table_S4.doc]

Supplementary Table 4 – Adonis (Permanova) results of benthic cover based on Bray-Curtis distances with 999 permutations. MS, mean sum of squares; SS, sum of squares.

|  | DF | SS | MS | Pseudo F | R^2^ | P value |
| --- | --- | --- | --- | --- | --- | --- |
| Site | 3 | 16.2179 | 5.406 | 129.65 | 0.58705 | 0.001*** |
| Year | 1 | 0.1397 | 0.1397 | 3.35 | 0.00506 | 0.046* |
| Site:Year | 3 | 1.4699 | 0.49 | 11.751 | 0.05321 | 0.001*** |
| Residuals | 235 | 9.7987 | 0.0417 |  | 0.35469 |  |
| Total | 242 | 27.6262 |  |  | 1 |  |
