# Supplementary material for: Metagenomics of Coral Reefs Under Phase Shift and High Hydrodynamics
Source: Front Microbiol. 2018 Oct 4;9:2203. doi: 10.3389/fmicb.2018.02203 (PMC6180206; doi:10.3389/fmicb.2018.02203)
Supplement: TABLE S5 — ANOVA results of total fish abundance. DF, degrees of freedom; SS, sum of squares; MS, mean sum of squares. [file Table_S5.doc]

Supplementary Table 5 – ANOVA results of total fish abundance. DF, degrees of freedom; SS, sum of squares; MS, mean sum of squares.

|  | DF | SS | MS | F value | P value |
| --- | --- | --- | --- | --- | --- |
| Site | 3 | 21.254 | 7.085 | 22.272 | 0.000 |
| Year | 1 | 0.129 | 0.129 | 0.407 | 0.526 |
| Site:Year | 3 | 2.269 | 0.756 | 2.378 | 0.077 |
| Residuals | 75 | 23.858 | 0.318 |  |  |
