# Supplementary material for: Metagenomics of Coral Reefs Under Phase Shift and High Hydrodynamics
Source: Front Microbiol. 2018 Oct 4;9:2203. doi: 10.3389/fmicb.2018.02203 (PMC6180206; doi:10.3389/fmicb.2018.02203)
Supplement: TABLE S6 — Adonis (Permanova) results of fish community abundance based on Bray-Curtis distances with 999 permutations. DF, degrees of freedom; SS, sum of squares; MS, mean sum of squares. [file Table_S6.doc]

Supplementary Table 6 – Adonis (Permanova) results of fish community abundance based on Bray-Curtis distances with 999 permutations. DF, degrees of freedom; SS, sum of squares; MS, mean sum of squares.

|  | DF | SS | MS | Pseudo F | R^2^ | P value |
| --- | --- | --- | --- | --- | --- | --- |
| Site | 3 | 3.5095 | 1.16984 | 8.7121 | 0.30139 | 0.001*** |
| Year | 1 | 0.8123 | 0.81227 | 6.0492 | 0.06975 | 0.001*** |
| Site:Year | 3 | 1.4146 | 0.47155 | 3.5117 | 0.12148 | 0.001*** |
| Residuals | 44 | 5.9082 | 0.13428 | 0.50738 |  |  |
| Total | 51 | 11.6446 | 1 |  |  |  |
