# Supplementary material for: Metagenomics of Coral Reefs Under Phase Shift and High Hydrodynamics
Source: Front Microbiol. 2018 Oct 4;9:2203. doi: 10.3389/fmicb.2018.02203 (PMC6180206; doi:10.3389/fmicb.2018.02203)
Supplement: TABLE S7 — ANOVA results of total bacterial abundance. DF, degrees of freedom; SS, sum of squares; MS, mean sum of squares. [file Table_S7.doc]

Supplementary Table 7 – ANOVA results of total bacterial abundance. DF, degrees of freedom; SS, sum of squares; MS, mean sum of squares.

|  |  | DF | SS | MS | F value | P value |
| --- | --- | --- | --- | --- | --- | --- |
| Bacterial Counts | Site | 3 | 1.25E+11 | 4.16E+10 | 21.42 | 1.22E-08 |
|  | Year | 1 | 4.91E+10 | 4.91E+10 | 25.3 | 9.17E-06 |
|  | Site:Year | 3 | 5.84E+10 | 1.95E+10 | 10.03 | 3.93E-05 |
|  | Residuals | 43 | 8.35E+10 | 1.94E+09 |  |  |
