# Supplementary material for: Metagenomics of Coral Reefs Under Phase Shift and High Hydrodynamics
Source: Front Microbiol. 2018 Oct 4;9:2203. doi: 10.3389/fmicb.2018.02203 (PMC6180206; doi:10.3389/fmicb.2018.02203)
Supplement: TABLE S8 — ANOVA results of Vibrio counts. DF, degrees of freedom; SS, sum of squares; MS, mean sum of squares. [file Table_S8.doc]

Supplementary Table 8 – ANOVA results of *Vibrio* counts. DF, degrees of freedom; SS, sum of squares; MS, mean sum of squares.

|  | DF | SS | MS | F value | P value |
| --- | --- | --- | --- | --- | --- |
| Site | 3 | 19324 | 6441 | 3.746 | 0.01461 |
| Year | 1 | 515 | 515 | 0.299 | 0.58604 |
| Site:Year | 3 | 35634 | 11878 | 6.907 | 0.00037 |
| Residuals | 73 | 125533 | 1720 |  |  |
