# Supplementary material for: Metagenomics of Coral Reefs Under Phase Shift and High Hydrodynamics
Source: Front Microbiol. 2018 Oct 4;9:2203. doi: 10.3389/fmicb.2018.02203 (PMC6180206; doi:10.3389/fmicb.2018.02203)
Supplement: TABLE S9 — ANOVA results of nutrients and chlorophyll a concentrations. DF, degrees of freedom; SS, sum of squares; MS, mean sum of squares. [file Table_S9.doc]

Supplementary Table 9 – ANOVA results of nutrients and chlorophyll a concentrations. DF, degrees of freedom; SS, sum of squares; MS, mean sum of squares.

|  |  | DF | SS | MS | F value | P value |
| --- | --- | --- | --- | --- | --- | --- |
| NO_2_ | Site | 3 | 0.00392 | 0.001307 | 0.621 | 0.6082 |
|  | Year | 1 | 0.01743 | 0.017433 | 8.278 | 0.0081 |
|  | Site:Year | 3 | 0.00309 | 0.001031 | 0.49 | 0.6926 |
|  | Residuals | 25 | 0.05265 | 0.002106 |  |  |
| NO_3_ | Site | 3 | 4.464 | 1.4881 | 3.989 | 0.0189 |
|  | Year | 1 | 0.162 | 0.1625 | 0.436 | 0.5153 |
|  | Site:Year | 3 | 0.986 | 0.3285 | 0.881 | 0.4645 |
|  | Residuals | 25 | 9.327 | 0.3731 |  |  |
| NH_4_ | Site | 3 | 6.12 | 2.04 | 1.021 | 0.400208 |
|  | Year | 1 | 42.52 | 42.52 | 21.265 | 0.000102 |
|  | Site:Year | 3 | 5.13 | 1.71 | 0.855 | 0.477135 |
|  | Residuals | 25 | 49.99 | 2 |  |  |
| PO_4_ | Site | 3 | 0.002165 | 0.0007215 | 0.709 | 0.5558 |
|  | Year | 1 | 0.000495 | 0.0004945 | 0.486 | 0.4922 |
|  | Site:Year | 3 | 0.007752 | 0.002584 | 2.538 | 0.0794 |
|  | Residuals | 25 | 0.025448 | 0.0010179 |  |  |
| SiO_2_ | Site | 3 | 0.0226 | 0.00752 | 0.256 | 0.8564 |
|  | Year | 1 | 0.1546 | 0.15458 | 5.262 | 0.0305 |
|  | Site:Year | 3 | 0.1108 | 0.03693 | 1.257 | 0.3105 |
|  | Residuals | 25 | 0.7344 | 0.02938 |  |  |
| Chlorophyll a | Site | 3 | 3.74 | 1.2468 | 0.912 | 0.457 |
|  | Year | 1 | 0.382 | 0.3818 | 0.279 | 0.604 |
|  | Site:Year | 3 | 7.563 | 2.521 | 1.844 | 0.18 |
|  | Residuals | 16 | 21.869 | 1.3668 |  |  |
