# Supplementary material for: Metagenomics of Coral Reefs Under Phase Shift and High Hydrodynamics
Source: Front Microbiol. 2018 Oct 4;9:2203. doi: 10.3389/fmicb.2018.02203 (PMC6180206; doi:10.3389/fmicb.2018.02203)
Supplement: TABLE S10 — Adonis (Permanova) results of metagenomic class composition (arcsin transformed) abundance based on Bray-Curtis distances with 999 permutations. MS, mean sum of squares; SS, sum of squares. [file Table_S10.doc]

Supplementary Table 10 – Adonis (Permanova) results of metagenomic class composition (arcsin transformed) abundance based on Bray-Curtis distances with 999 permutations. MS, mean sum of squares; SS, sum of squares.

|  | Df | SS | MS | Pseudo-F | R^2^ | P value |
| --- | --- | --- | --- | --- | --- | --- |
| Site | 3 | 0.05006 | 0.0166867 | 1.8462 | 0.36802 | 0.308 |
| Year | 1 | 0.014764 | 0.0147644 | 1.6335 | 0.10854 | 0.332 |
| Site:Year | 2 | 0.053123 | 0.0265615 | 2.9387 | 0.39054 | 0.186 |
| Residuals | 2 | 0.018077 | 0.0090385 |  | 0.13289 |  |
| Total | 8 | 0.136025 |  |  | 1 |  |
