# Supplementary material for: Metagenomics of Coral Reefs Under Phase Shift and High Hydrodynamics
Source: Front Microbiol. 2018 Oct 4;9:2203. doi: 10.3389/fmicb.2018.02203 (PMC6180206; doi:10.3389/fmicb.2018.02203)
Supplement: TABLE S11 — Adonis (Permanova) results of metagenomic level 1 subsystem composition (arcsin transformed) abundance based on Bray-Curtis distances with 999 permutations. MS, mean sum of squares; SS, sum of squares. [file Table_S11.doc]

Supplementary Table 11 – Adonis (Permanova) results of metagenomic level 1 subsystem composition (arcsin transformed) abundance based on Bray-Curtis distances with 999 permutations. MS, mean sum of squares; SS, sum of squares.

|  | DF | SS | MS | Pseudo F | R^2^ | P value |
| --- | --- | --- | --- | --- | --- | --- |
| Site | 3 | 0.0011299 | 0.00037664 | 0.77288 | 0.28496 | 0.701 |
| Year | 1 | 0.0013428 | 0.00134281 | 2.75551 | 0.33865 | 0.075 |
| Site:Year | 2 | 0.0005178 | 0.00025889 | 0.53125 | 0.13058 | 0.862 |
| Residuals | 2 | 0.0009746 | 0.00048732 | 0.2458 |  |  |
| Total | 8 | 0.0039651 | 1 |  |  |  |
