# Supplementary material for: Metagenomics of Coral Reefs Under Phase Shift and High Hydrodynamics
Source: Front Microbiol. 2018 Oct 4;9:2203. doi: 10.3389/fmicb.2018.02203 (PMC6180206; doi:10.3389/fmicb.2018.02203)
Supplement: TABLE S12 — Adonis (Permanova) results of metagenomic dinucleotides composition abundance based on Bray-Curtis distances with 999 permutations. MS, mean sum of squares; SS, sum of squares. [file Table_S12.doc]

Supplementary Table 12 – Adonis (Permanova) results of metagenomic dinucleotides composition abundance based on Bray-Curtis distances with 999 permutations. MS, mean sum of squares; SS, sum of squares.

|  | DF | SS | MS | Pseudo F | R^2^ | P value |
| --- | --- | --- | --- | --- | --- | --- |
| Site | 3 | 0.045426 | 0.0151419 | 2.97848 | 0.6151 | 0.036* |
| Year | 1 | 0.002992 | 0.0029916 | 0.58847 | 0.04051 | 0.723 |
| Site:Year | 2 | 0.015266 | 0.0076328 | 1.50141 | 0.20671 | 0.293 |
| Residuals | 2 | 0.010168 | 0.0050838 | 0.13768 |  |  |
| Total | 8 | 0.073851 | 1 |  |  |  |
