# Supplementary material for: Metagenomics of Coral Reefs Under Phase Shift and High Hydrodynamics
Source: Front Microbiol. 2018 Oct 4;9:2203. doi: 10.3389/fmicb.2018.02203 (PMC6180206; doi:10.3389/fmicb.2018.02203)
Supplement: TABLE S13 — Wave characteristics extracted based on typhoon wave conditions. Hs: significant wave height (m); Dir: direction in degrees North; P: wave power (W/m). [file Table_S13.doc]

Supplementary Table 13 – Wave characteristics extracted based on typhoon wave conditions. Hs: significant wave height (m); Dir: direction in degrees North; P: wave power (W/m).

| **Typhoon condition** | | |  |  |
| --- | --- | --- | --- | --- |
|  | **Hs** | **dir** | **T** | **P (W/m)** |
| **Offshore** | **12.0** | **106** | **14** | **1982985** |
| Taketomi | 2.6 | 163 | 4 | 29628 |
| Sekisei | 4.0 | 147 | 6 | 92100 |
| Osaki | 4.8 | 170 | 7 | 168515 |
| Miyara | 9.9 | 135 | 14 | 1319518 |
| **Calm condition** | |  |  |  |
|  | **Hs** | **dir** | **T** | **P (W/m)** |
| **Offshore** | **1.5** | **100** | **8** | **17705** |
| Taketomi | 0.3 | 130 | 8 | 708 |
| Sekisei | 0.3 | 128 | 8 | 708 |
| Osaki | 0.4 | 179 | 8 | 1259 |
| Miyara | 1.2 | 103 | 8 | 11331 |
